# Clinical Applications and Decision-Making Impact of Contrast-Enhanced Mammography and MRI: A Recent Structured Review

**DOI:** 10.3390/diagnostics16131987

**Published:** 2026-06-26

**Authors:** Norhayati Mohd Zain, Wan Azani Mustafa

**Affiliations:** 1 Medical Imaging Department, School of Integrative Medicine & Life Sciences, KPJ Healthcare University, Nilai 71800, Negeri Sembilan, Malaysia; 2 Faculty of Electrical Engineering & Technology, Universiti Malaysia Perlis, Pauh Putra Campus, Arau 02600, Perlis, Malaysia; 3 Advanced Computing (AdvCOMP), Centre of Excellence, Universiti Malaysia Perlis, Pauh Putra Campus, Arau 02600, Perlis, Malaysia

**Keywords:** breast cancer imaging, diagnostic accuracy, diagnostic performance

## Abstract

Contrast-Enhanced Mammography (CEM) and breast Magnetic Resonance Imaging (MRI) have emerged as important advanced imaging modalities that extend beyond conventional mammography by improving lesion visualization, diagnostic confidence, and clinical decision-making in breast cancer management. However, variability in their clinical application, comparative performance, and impact on workflow and healthcare costs remains a challenge for evidence-based implementation. This structured thematic review aimed to synthesize recent evidence on the clinical applications and decision-making impact of CEM and MRI in breast imaging practice. The review was conducted in accordance with the Preferred Reporting Items for Systematic Reviews and Meta-Analyses (PRISMA) guidelines. A comprehensive literature search was performed using Scopus and PubMed databases, employing advanced keyword combinations including “contrast-enhanced mammography” and “magnetic resonance imaging.” Following systematic screening, eligibility assessment, and quality appraisal, 20 Primary Studies (PS) were included in the final qualitative synthesis. The findings were organized into three thematic domains: (1) diagnostic accuracy and comparative performance, (2) clinical implementation and workflow of CEM, and (3) cost-effectiveness and clinical decision impact. Across studies, CEM demonstrated diagnostic accuracy comparable to MRI for lesion detection and characterization, particularly in dense breast tissue, while offering advantages in accessibility, examination time, and patient tolerance. Evidence also highlighted the growing role of CEM in preoperative assessment, treatment monitoring, and problem-solving scenarios, with favorable implications for workflow efficiency. Furthermore, several studies reported potential cost savings and improved clinical decision-making when CEM was used as an alternative or complementary modality to MRI. In conclusion, current evidence supports the expanding clinical role of CEM alongside MRI, highlighting its value as a diagnostically robust imaging modality with potential economic and workflow advantages in selected clinical settings.

## 1. Introduction

Breast cancer remains the most prevalent malignancy among women worldwide, necessitating early and accurate detection to improve outcomes. Conventional mammography, while widely utilized, is limited by reduced sensitivity in dense breast tissue and can yield high false-positive and false-negative rates, especially in younger women and those with complex breast architecture [1,2]. Thus, to address these challenges, Contrast-Enhanced (CE) imaging techniques have been developed. CEM combines dual-energy mammography with intravenous iodinated contrast, enhancing the visualization of tumor neovascularity and providing both morphological and functional data [2,3,4,5,6,7,8,9]. Breast MRI, particularly with dynamic contrast enhancement, is recognized for its superior sensitivity in detecting invasive cancers and its utility in high-risk populations, preoperative staging, and treatment monitoring [1,10]. The integration of these modalities into breast cancer screening and diagnostic pathways is reshaping personalized care, with ongoing research evaluating their comparative effectiveness, accessibility, and cost-efficiency [3,4,5,6,11,12].

Contrast-Enhanced Mammography (CEM) and breast Magnetic Resonance Imaging (MRI) are advanced imaging modalities that have transformed the landscape of breast cancer detection, diagnosis, and management. Both techniques utilize contrast agents to highlight vascularity and tissue characteristics associated with malignancy, offering functional and anatomical information beyond conventional mammography. The increasing incidence of breast cancer and the limitations of traditional imaging, particularly in women with dense breast tissue, have driven the adoption and ongoing evaluation of these modalities in clinical practice [1,2,8].

Recent literature underscores the high diagnostic performance of CEM, with pooled sensitivities ranging from 89% to 97.7% and specificities between 50% and 89% in dense breasts, closely approaching the accuracy of breast MRI [2,13,14,15]. Furthermore, CEM’s dual-energy approach allows for the detection of both anatomical abnormalities and functional changes, such as neovascularity, which are hallmarks of malignancy [2,7,8]. Studies have established that CEM outperforms conventional mammography and ultrasound, particularly in women with dense breasts or inconclusive findings, and has been proposed as a potentially more accessible and economically favorable alternative to MRI in selected clinical settings, although direct cost-effectiveness evidence remains limited [2,7,13,16,17,18]. Additionally, CEM has demonstrated promise in preoperative staging, monitoring response to neoadjuvant therapy, and reducing unnecessary biopsies due to its high negative predictive value [13,19].

Breast MRI remains the gold standard for sensitivity, especially in high-risk women and those with dense breast tissue, with reported sensitivities up to 100% in some screening cohorts [1,10]. MRI is invaluable for assessing disease extent, evaluating response to therapy, and detecting occult lesions not visible on other modalities [1,10]. However, its higher cost, limited availability, and contraindications in certain patient populations restrict its universal application. Recent advances in abbreviated MRI protocols and the integration of artificial intelligence (AI) and radiomics are being explored to enhance efficiency, reduce costs, and improve diagnostic accuracy [1,20,21].

Comparative studies and meta-analyses published in the last five years highlight that while CEM and MRI both offer high sensitivity, CEM provides greater accessibility, lower cost, and better patient tolerance, with only a slight increase in radiation dose and a low risk of contrast-related adverse events [2,7,8,14]. CEM is particularly advantageous in resource-constrained settings and for patients who cannot undergo MRI due to contraindications or intolerance [2,7,8]. Nevertheless, MRI maintains a role in complex cases, such as evaluating multifocal or multicentric disease, and in populations at very high risk [1,10].

Emerging research also highlights the potential of radiomics and deep learning applications in both CEM and MRI, aiming to extract quantitative imaging biomarkers that may further refine risk stratification, prognostication, and individualized treatment planning [20,22,23,24,25,26]. These technological advancements are expected to bridge current gaps in diagnostic performance and workflow efficiency, supporting the broader integration of CE imaging in breast cancer care.

In summary, both CEM and breast MRI have established themselves as pivotal tools in modern breast imaging. The literature from the past five years consistently demonstrates their complementary roles, with CEM offering a practical, high-performance alternative to MRI in many scenarios, and MRI retaining its status as the most sensitive modality for comprehensive breast cancer assessment [1,2,7,8,10,14]. Concurrently, ongoing research and technological innovation continue to refine their applications, promising further improvements in early detection, diagnosis, and personalized management of breast cancer.

## 2. Research Question

This is a PRISMA-guided structured narrative review with qualitative thematic synthesis, where Research Questions (RQs) serve as the foundation that guides the entire review process. They define the scope and objectives of the study, directly shaping the selection and exclusion of publications to ensure relevance and methodological rigor. Well-defined and focused questions enable the development of a structured and comprehensive search strategy, helping to capture all relevant evidence while minimizing bias. RQs also provide a clear framework for data extraction, analysis, and synthesis, ensuring that findings are organized and presented in a coherent manner. Thus, by establishing clear boundaries, they maintain the focus of the review and prevent deviation from its intended purpose. Moreover, clearly articulated questions enhance transparency and reproducibility, allowing other researchers to replicate the review or build upon its findings. Overall, robust RQs give an SLR its direction and significance, whether the aim is to identify research gaps, evaluate intervention outcomes, or examine emerging trends.

Specifying the RQs is the most crucial task in the planning phase of an SLR and remains central throughout the review process, as they guide and shape the overall methodology [27]. Since this SLR seeks to explore and evaluate recent advancements within the selected domain, a well-defined and systematic structure is essential to ensure a clear and focused direction. Accordingly, the PICo framework was adopted to guide the development of the RQs, following the recommendations of Lockwood et al. [28]. PICo, which is particularly suited for qualitative research, comprises three key components (Population, Interest, and Context), facilitating the formulation of precise and well-aligned RQs.

Within the PICo framework, the Population (P) refers to the specific individuals or groups under investigation, such as a defined demographic, patient subgroup, or wider community. The Interest (I) represents the main phenomenon or focus of the study, which may include particular experiences, behaviors, interventions, or issues of concern. The Context (Co) describes the setting in which the research takes place, encompassing factors such as geographical location, cultural background, or social environment. Applying the PICo framework enables RQs to be developed in a structured and systematic manner. This ensures that all key components of the study are clearly defined, supports a more targeted and efficient literature search, and enhances the overall clarity and coherence of the SLR. Based on this framework, the present study formulated one RQ, as outlined below:

1. RQ1: In women undergoing diagnostic or preoperative breast imaging (P), how does CEM (I) compare to CE breast MRI (CE-MRI) and other imaging modalities in terms of diagnostic accuracy, lesion detection, and tumor size or extent estimation (Co) during the clinical diagnostic workup or preoperative staging of breast cancer?
2. RQ2: In radiology practices and imaging centers (P), what are the main operational barriers and facilitators of implementing CEM (I) in routine clinical or screening settings, including patient selection, workflow integration, contrast administration, and resource allocation (Co)?
3. RQ3: Among patients with suspicious breast lesions and healthcare decision-makers (P), how does the use of CEM or CE-MRI (I) affect cost-effectiveness and influence surgical planning and biopsy decisions (Co) within preoperative or diagnostic pathways for breast cancer evaluation?

## 3. Materials and Methods

The Preferred Reporting Items for Systematic Reviews and Meta-Analyses (PRISMA) framework, as outlined by Page et al. [29], is broadly recognized as a standard protocol for performing structured reviews. Its purpose is to promote a clear, thorough, and structured review process. By adhering to PRISMA, researchers enhance the reliability and credibility of their findings through well-defined steps that involve locating, evaluating, and selecting studies relevant to the research focus. The framework also underscores the role of randomized research in minimizing bias and strengthening the overall quality of evidence. In this SLR, Scopus and PubMed were selected as the primary sources of literature due to their broad scientific coverage and established authority as trusted databases.

The PRISMA workflow is organized into four essential phases: identification, screening, eligibility, and data extraction. In the identification phase, a comprehensive search is performed across selected databases to gather all studies that may be relevant to the topic. The screening phase then applies predefined inclusion and exclusion criteria to remove publications that do not align with the review’s objectives or quality standards. Following this, the eligibility phase involves a closer evaluation of the remaining articles to confirm that they fully satisfy the criteria for inclusion. Finally, during the data extraction phase, pertinent information from the selected studies is collected, categorized, and synthesized to draw meaningful conclusions. This systematic process enhances research quality and transparency, ensuring that the final review presents trustworthy evidence to support ongoing scholarship and inform professional practice. To improve the comprehensiveness of the literature search, a Generative Artificial Intelligence (GenAI) tool was utilized during the keyword identification phase. The tool was used to generate synonyms, related terms, and alternative expressions for the main research concepts. These suggested terms were subsequently reviewed and validated by the authors before being incorporated into the final search strings. The use of GenAI facilitated the development of a broader and more systematic search strategy, thereby enhancing the retrieval of relevant publications from the selected databases. Overall, a total of 4463 records were identified through database searching (Scopus = 3658; PubMed = 805). Following duplicate removal and application of predefined screening criteria, 171 records were retained for full-text assessment. During the eligibility phase, 151 studies were excluded due to lack of relevance to the review objectives, absence of empirical findings, incomplete full-text availability, or failure to meet inclusion criteria. Ultimately, 20 studies were included in the qualitative synthesis.

### 3.1. Identification

In accordance with the PRISMA framework, the identification phase serves as the foundational step in ensuring a structured and transparent approach to evidence retrieval. Using PRISMA-guided search strategies, Scopus and PubMed were selected as the primary databases due to their comprehensive indexing of high-quality and peer-reviewed biomedical literature. Through systematic keyword searching focused on “contrast-enhanced mammography” and “magnetic resonance imaging,” a total of 4463 records were identified, 3658 from Scopus and 805 from PubMed as shown in Table 1. This substantial dataset highlights the rapidly expanding body of research dedicated to advanced breast imaging modalities, reflecting global efforts to enhance early detection and diagnostic precision in breast cancer management. The high volume of publications retrieved illustrates the growing clinical interest and technological advancements supporting the integration of CEM and MRI in routine imaging pathways.

Furthermore, the substantial number of identified studies provides strong justification for applying subsequent PRISMA phases, specifically screening, eligibility, and inclusion, to refine the evidence base and minimize bias. While the large dataset signifies robust academic engagement, it also indicates significant heterogeneity in study populations, clinical objectives, and imaging protocols. This underscores the necessity of a structured review process to evaluate methodological quality, eliminate duplications, and synthesize the most relevant findings that can inform clinical guidelines and future innovation. The application of the PRISMA framework, therefore, ensures methodological rigor while also strengthening the reliability and transparency of the review outcomes in advancing evidence-based breast imaging practices.

**Table 1:** The Search String.

Scopus	TITLE-ABS-KEY ((contrast OR enhance) AND (mammogram OR mammography) AND (magnetic resonance imaging OR MRI)) AND PUBYEAR > 2024 AND PUBYEAR < 2026 AND (LIMIT-TO (SRCTYPE, “j”)) AND (LIMIT-TO (PUBSTAGE, “final”)) AND (LIMIT-TO (EXACTKEYWORD, “Breast Magnetic Resonance Imaging”) OR LIMIT-TO (EXACTKEYWORD, “Contrast Enhanced Mammography”) OR LIMIT-TO (EXACTKEYWORD, “Contrast-enhanced Mammography”)) AND (LIMIT-TO (DOCTYPE, “ar”)) AND (LIMIT-TO (SUBJAREA, “MEDI”)) AND (LIMIT-TO (LANGUAGE, “English”)) Date of Access: December 2025
PubMed	Search: (contrast OR enhance) AND (mammogram OR mammography) AND (MRI OR magnetic resonance imaging) Filters: Abstract, Free full text, Full text, Adaptive Clinical Trial, Address, Autobiography, Bibliography, Biography, Books and Documents, Case Reports, Classical Article, Clinical Conference, Clinical Study, Clinical Trial, Clinical Trial, Phase I, Clinical Trial, Phase II, Clinical Trial, Phase III, Clinical Trial, Phase IV, Clinical Trial Protocol, Collected Work, Comment, Comparative Study, Congress, Consensus Development Conference, Consensus Development Conference, NIH, Controlled Clinical Trial, Corrected and Republished Article, Dataset, Dictionary, Directory, Duplicate Publication, Editorial, Electronic Supplementary Materials, English Abstract, Equivalence Trial, Evaluation Study, Expression of Concern, Festschrift, Government Publication, Guideline, Historical Article, Interactive Tutorial, Interview, Introductory Journal Article, Lecture, Legal Case, Legislation, Letter, Meta-Analysis, Multicenter Study, Network Meta-Analysis, News, Newspaper Article, Observational Study, Overall, Patient Education Handout, Periodical Index, Personal Narrative, Portrait, Practice Guideline, Pragmatic Clinical Trial, Preprint, Published Erratum, Randomized Controlled Trial, Randomized Controlled Trial, Veterinary, Research Support, American Recovery and Reinvestment Act, Research Support, NIH, Extramural, Research Support, NIH, Intramural, Research Support, Non-US Gov’t, Research Support, US Gov’t, Research Support, US Gov’t, Non-PHS, Research Support, US Gov’t, PHS, Retracted Publication, Retraction of Publication, Clinical Trial, Veterinary, Observational Study, Veterinary, English, from 1 January 2025–30 November 2025 Date of Access: December 2025

### 3.2. Screening

In the screening stage of this review, the studies collected from the initial search were thoroughly evaluated for their relevance and quality. Out of the 4463 records identified during the identification phase, a total of 171 articles were shortlisted after the initial screening process. This selection followed predetermined inclusion and exclusion criteria to ensure that only publications directly related to the review’s aims were retained (Table 2). Titles and abstracts were assessed to confirm their relevance to CEM, and breast MRI was conducted independently by two reviewers. Studies considered potentially relevant by either reviewer proceeded to full-text assessment. Any disagreements regarding inclusion were resolved through discussion and consensus. Where consensus could not initially be reached, a third reviewer acted as an adjudicator. Formal blinding of reviewers was not performed because study identifiers and publication details were visible during screening. In addition, duplicate entries were removed to maintain data consistency and reliability.

A total of 4279 records were subsequently excluded based on predefined criteria aimed at strengthening methodological quality and ensuring that only credible, peer-reviewed research was considered. Studies were removed if they were published before 2025, written in languages other than English, or classified as reviews, book chapters, conference papers, or articles still “in press.” Through this filtering process, only original studies with complete and verifiable data were retained, in alignment with PRISMA guidelines. The substantial number of exclusions demonstrates the significance of applying strict criteria within a rapidly advancing research area, where secondary and non-validated publications are increasingly prevalent. As a result, this step produced a more focused, reliable, and language-consistent dataset that serves as a solid foundation for the following eligibility and inclusion stages of the review.

In addition, full-text analysis was conducted for all studies included after the screening phase to ensure a more comprehensive and accurate eligibility assessment beyond title and abstract review. This step allowed for a more detailed evaluation of methodological quality and relevance to the review objectives. The substantial number of exclusions demonstrates the importance of applying strict criteria within a rapidly advancing research area, where secondary and non-validated publications are increasingly prevalent. As a result, this process produced a more focused, reliable, and language-consistent dataset that serves as a solid foundation for the subsequent eligibility and inclusion stages of the review. The findings derived from this synthesis should be interpreted as indicative rather than definitive, reflecting the evolving nature of evidence in this field.

### 3.3. Eligibility

During the eligibility stage, all 171 shortlisted articles underwent a detailed evaluation. The titles, abstracts, and full-text articles were systematically assessed to determine their eligibility and relevance to the objectives of this review. Full-text screening was independently conducted by two reviewers using predefined inclusion and exclusion criteria. Each reviewer evaluated the studies separately to minimize selection bias and ensure consistency in the screening process. Any discrepancies regarding study eligibility were resolved through discussion and consensus. Where necessary, disagreements were further reviewed until a final agreement was reached. This structured approach enhanced the transparency, reliability, and reproducibility of the study selection process. Throughout this assessment, 151 studies were excluded as they fell outside the scope of the topic, lacked alignment with the research focus, did not provide full-text access, or failed to include empirical findings. Consequently, a total of 20 articles fulfilled all required criteria and were included in the final stage of the structured thematic review.

### 3.4. Data Abstraction and Analysis

**Figure 1:**
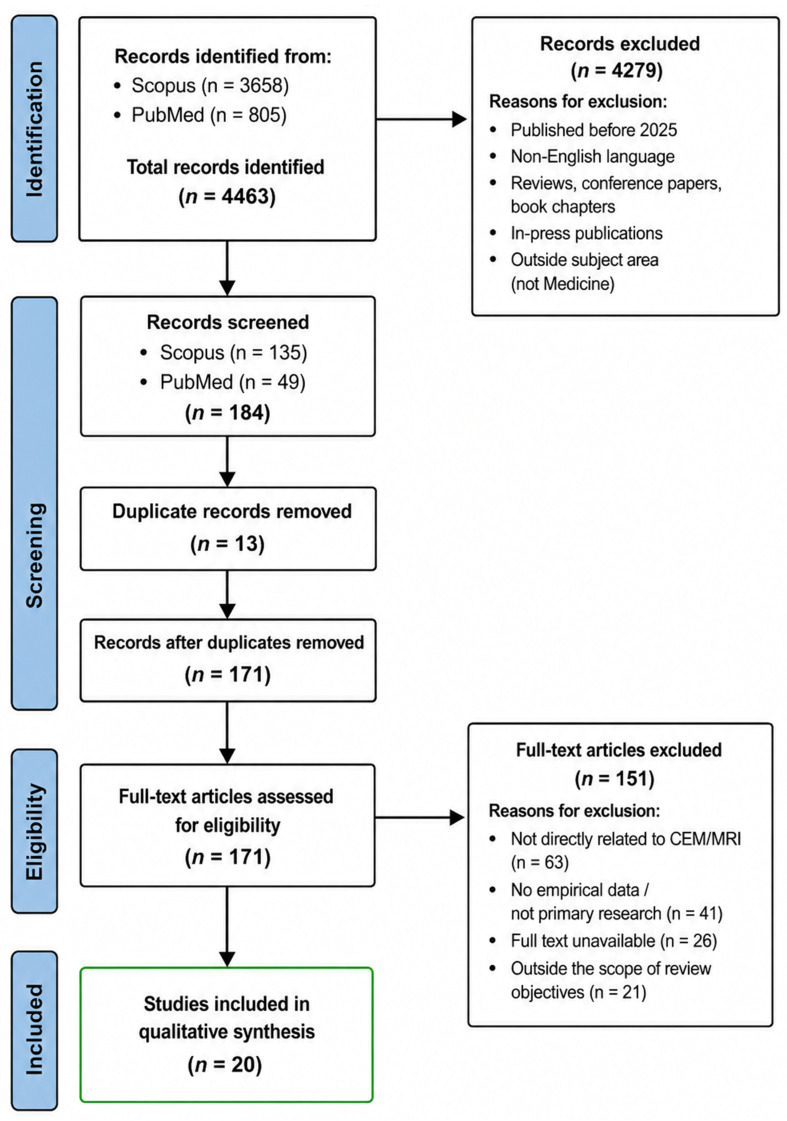
Flow diagram of the proposed searching study.

An integrative analysis method was applied to synthesize evidence from various research designs, with emphasis on qualitative studies. The main objective of this approach was to identify important themes and subthemes relevant to the RQs. The process began with data extraction, serving as the foundation for theme development. As illustrated in Figure 1, a total of 20 eligible studies (Table 3) were thoroughly examined to identify statements and findings related to the aims of this review. The authors assessed the key contributions of these studies, focusing on CEM and breast MRI, including an evaluation of their methodologies and reported outcomes. Collaboration among the authors ensured that emerging themes were firmly supported by the extracted evidence. Throughout the analysis, a detailed log was maintained to record insights, reflections, and interpretive notes. Finally, the authors cross-checked the results for accuracy and coherence in theme formulation. Any differing viewpoints were discussed collectively to reach an agreement and ensure consistency in the overall synthesis.

### 3.5. Quality of Appraisal

In accordance with the guidelines proposed by Kitchenham and Charters [27], once the Primary Studies (PSs) have been selected, their research quality must be assessed and a quantitative comparison conducted. In this study, the Quality Assessment (QA) method introduced by Abouzahra et al. [50] was adopted, which comprises six QA criteria for the SLR. The selected studies included diagnostic accuracy investigations, implementation studies, screening studies, economic evaluations, and qualitative research. Therefore, a generic methodological appraisal framework was selected to allow consistent assessment across heterogeneous study designs. Each criterion was evaluated using a three-point scoring scale: “Yes” (Y), assigned a score of 1 when the criterion was fully satisfied; “Partly” (P), assigned a score of 0.5 when it was partially met with minor limitations; and “No” (N), assigned a score of 0 when the criterion was not satisfied. The six-QA criteria applied in this study are defined as follows:

QA1. Is the purpose of the study clearly stated?

QA2. Are the interest and the usefulness of the work clearly presented?

QA3. Is the study methodology clearly established?

QA4. Are the concepts of the approach clearly defined?

QA5. Is the work compared and measured with other similar work?

QA6. Are the limitations of the work clearly mentioned?

The table summarizes the QA procedure used to evaluate each selected study against predefined criteria. Three experts independently assessed the studies, assigning one of three ratings for each criterion: “Yes” (Y) when the criterion was fully met, “Partly” (P) when it was partially satisfied with minor limitations, or “No” (N) when it was not met. Each expert conducted the evaluation independently, and the individual scores were subsequently aggregated to obtain a total QA score for each study. To be included in the subsequent stage of the review, a study was required to achieve a cumulative score exceeding 3.0. This threshold ensured that only studies of acceptable methodological quality were retained for further analysis.

## 4. Results and Discussion

Based on the findings presented in Table 4, the overall methodological quality of the selected PSs related to CEM and breast MRI is generally satisfactory. Most studies achieved a total mark of 4.5 (75%), demonstrating strong adherence to essential quality appraisal criteria such as clearly stated objectives, appropriate study designs, and reliable data reporting. This consistent performance indicates that the research on CEM and breast MRI included in this review meets acceptable scientific standards and contributes credible evidence to the field. Only two studies, PS4 and PS18, scored slightly lower (4.0; 66.7%), reflecting minor methodological limitations. Importantly, no study was classified as low quality, reinforcing confidence in the reliability of the selected literature.

The assessment further highlights that, while the majority of studies conducted in the domain of CEM and breast MRI exhibit sound research integrity, there remain opportunities for improvement, particularly in areas such as sample diversity, detailed methodological transparency, and clearly defined inclusion criteria. Nonetheless, the predominance of high-quality ratings supports the robustness of this systematic analysis and strengthens the validity of the synthesized findings regarding the diagnostic value and clinical advancements of CEM compared to breast MRI. The relatively narrow range of QA scores reflects the strict eligibility criteria applied during study selection, which restricted inclusion to recent peer-reviewed journal articles meeting predefined methodological standards. Consequently, the appraisal served primarily as a quality verification procedure rather than a mechanism for ranking studies according to methodological superiority. In conclusion, the QA confirms that all twenty reviewed studies are suitable for inclusion, enabling this review to provide a comprehensive and trustworthy interpretation of current research trends and evidence in advanced breast imaging modalities (Table 5).

### 4.1. Diagnostic Accuracy and Comparative Performance

Results are presented according to thematic domains rather than study design, reflecting the substantial methodological heterogeneity among included studies. In order to enhance interpretability, the reviewed studies were stratified into key analytical subgroups based on clinical application and imaging performance domains, namely: (1) detection of multifocal/multicentric disease, (2) treatment response assessment, (3) lesion characterization and size estimation, (4) quantitative and scoring-based evaluation, and (5) lesion conspicuity and reader-dependent outcomes.

#### 4.1.1. Detection of Multifocal and Multicentric Disease

Within this subgroup, CEM demonstrates diagnostic performance comparable to CE-MRI, particularly in sensitivity. Gouda et al. [30] reported equivalent sensitivity and overall accuracy between the two modalities, with CEM showing superior specificity. This suggests that CEM may be more effective in reducing false-positive findings, which is a recognized limitation associated with MRI and can contribute to unnecessary follow-up investigations or biopsies. In this context, CEM may offer a more balanced diagnostic profile by maintaining high detection capability while improving specificity. Furthermore, Haggag et al. [31] highlighted that combining CEM with digital breast tomosynthesis (DBT) further improves detection capability, approaching the performance of dynamic CE-MRI. This combined approach appears to improve lesion visualization and diagnostic confidence, particularly in complex or subtle cases. Collectively, these findings indicate that while MRI remains the reference standard for comprehensive breast lesion detection, CEM, especially when integrated with DBT, represents a competitive and potentially more specific alternative within this subgroup, with promising implications for clinical workflow optimization.

#### 4.1.2. Assessment of Treatment Response (Neoadjuvant Therapy)

In evaluating tumor response to neoadjuvant therapy, Wang et al. [34] demonstrated that CEM achieves sensitivity comparable to MRI in detecting residual disease. However, a consistent limitation identified is the tendency of CEM to slightly overestimate residual tumor size, which may be attributed to persistent background parenchymal enhancement and the reliance on contrast uptake patterns. This overestimation could potentially influence treatment assessment and surgical decision-making if not carefully interpreted within the broader clinical context. In contrast, MRI provides more precise delineation of residual disease due to its superior soft tissue contrast and functional imaging capability, allowing for more accurate assessment of tumor boundaries and treatment response. Therefore, while CEM is clinically useful for monitoring therapeutic response and offers a practical alternative in many settings, MRI remains preferable when precise residual tumor measurement is critical for surgical planning and achieving optimal oncological outcomes.

#### 4.1.3. Lesion Characterization and Tumor Size Estimation

CEM shows clear advantages over conventional digital mammography in this subgroup. ElSayad et al. [32] reported higher specificity and improved correlation with histopathological tumor size, particularly in lesions associated with suspicious calcifications. This suggests that CEM may provide more reliable lesion characterization and size estimation in certain complex presentations, thereby enhancing diagnostic confidence and potentially improving preoperative assessment accuracy. However, modality-specific strengths remain evident. Ma et al. [35] found that contrast-enhanced cone-beam breast CT demonstrates high agreement with MRI in detecting both mass and non-mass lesions, whereas conventional mammography retains superiority in detecting fine calcifications. Additionally, Azcona Sáenz et al. [36] emphasized that tumor size estimation is most accurate when using CEM, MRI, or DBT in cases of architectural distortion without thin spicules. Collectively, these findings indicate that diagnostic accuracy is highly dependent on lesion type and imaging context, supporting a stratified and lesion-specific approach to modality selection rather than a one-size-fits-all strategy.

#### 4.1.4. Quantitative Analysis and Structured Scoring Systems

The integration of quantitative parameters and standardized scoring systems represents a growing area of advancement for CEM. Bülüç et al. [37] demonstrated that metrics such as relative signal difference and relative signal contrast strongly correlate with histopathological outcomes, enabling objective differentiation between benign and malignant lesions. Similarly, Hua et al. [42] showed that applying the Kaiser score to CEM images yields diagnostic accuracy comparable to MRI, suggesting that established MRI-based frameworks can be effectively translated to CEM. Johansson Lipinski et al. [43] further supported the utility of CEM in evaluating invasive lobular carcinoma, particularly in accurately determining disease extent. Despite these strengths, MRI still offers a broader range of functional and kinetic parameters, indicating a relative advantage in advanced quantitative imaging.

#### 4.1.5. Lesion Conspicuity and Reader Confidence

Reader-dependent factors, including lesion conspicuity and diagnostic confidence, form an important subgroup influencing clinical applicability. Clauser et al. [38] reported that although CE-MRI maintains the highest overall diagnostic accuracy, contrast-enhanced DBT provides superior lesion conspicuity and enhances reader confidence compared to standard CEM. This indicates that beyond purely diagnostic performance metrics, the perceived clarity of lesion visualization plays a crucial role in how effectively imaging findings are interpreted in clinical practice, particularly among radiologists with varying levels of experience. Similarly, Santonocito et al. [39] found that CE-MRI offers better lesion conspicuity, particularly for benign lesions, which may be less distinctly visualized on CEM. These findings suggest that visual clarity and interpretability may differ across modalities, even when diagnostic accuracy appears comparable. Taken together, these studies highlight that reader confidence and lesion visibility are modality-dependent factors that may influence reporting consistency, diagnostic decision-making, and ultimately the clinical utility of each imaging technique in routine breast imaging workflows.

### 4.2. Clinical Implementation and Workflow of CEM

The integration of CEM into breast cancer screening programs has demonstrated promising outcomes in improving cancer detection, though certain limitations remain. Berg et al. [40] reported findings from the Screening Contrast-Enhancement Mammography (SCEMAM) trial indicating that CEM, when used as a supplemental screening tool, increased cancer detection rates compared to conventional methods and achieved a higher Area Under the Receiver Operating Characteristic Curve (AUROC), reflecting improved diagnostic performance. However, this increase in sensitivity was accompanied by a higher false-positive rate, highlighting a trade-off between detection efficiency and specificity. In essence, these results suggest that while CEM can augment conventional screening strategies, careful patient selection and risk stratification are essential to maximize clinical benefits while minimizing unnecessary follow-up procedures.

Practical implementation of CEM in clinical settings involves multiple operational and logistical considerations. For instance, Dashevsky et al. [41] emphasized that barriers to routine adoption include challenges related to selecting appropriate patient populations, integrating CEM into existing imaging workflows, administering contrast agents safely, and navigating billing and reimbursement processes. The study further outlined strategies to address these obstacles, including workflow optimization, staff training, and clear guidelines for patient eligibility, underscoring that successful integration of CEM requires both technical adaptation and administrative planning. Collectively, these findings indicate that while CEM holds substantial potential as a screening adjunct and diagnostic tool, its widespread adoption necessitates structured implementation strategies and awareness of operational challenges.

### 4.3. Cost-Effectiveness and Clinical Decision Impact

While direct cost-effectiveness evidence for CEM remains limited among the included studies, its shorter examination time, wider availability, and integration into existing mammography infrastructure have been identified as factors that may contribute to lower overall healthcare costs compared with MRI in selected clinical scenarios. Fueger et al. [33] conducted an analysis comparing the cost-effectiveness of supplemental breast MRI with stereotactic biopsy, finding that both approaches resulted in comparable financial expenditures and clinical outcomes. The study highlighted that MRI could serve as a non-invasive alternative to biopsy in selected cases, potentially reducing procedural risks while maintaining diagnostic accuracy. This evidence suggests that incorporating non-invasive imaging options such as MRI may optimize resource utilization without compromising patient care outcomes.

The impact of CEM on surgical planning and clinical decisions has also been demonstrated. ElSayad et al. [32] reported that the use of CEM enabled more precise assessment of tumor size and detection of multicentric lesions, thereby informing surgical strategies more accurately. Hence, by improving lesion characterization and providing detailed anatomical information, CEM contributed to better-informed decisions regarding surgical margins, resection planning, and the extent of intervention required. This emphasizes the dual benefit of advanced imaging modalities: enhancing diagnostic precision while influencing treatment pathways in a clinically meaningful manner.

## 5. Conclusions

This structured thematic review provides a structured synthesis of current evidence on the comparative and complementary roles of contrast-enhanced mammography (CEM) and breast magnetic resonance imaging (MRI) in breast cancer detection and clinical management. Rather than identifying a single superior modality, the findings consistently demonstrate that diagnostic performance is highly context-dependent, varying according to lesion characteristics, clinical indication, and healthcare setting. Across the reviewed studies, CEM emerges as a clinically valuable modality with diagnostic accuracy approaching that of MRI, particularly in the detection of multifocal and multicentric disease, as well as in preoperative tumor size estimation. Its relatively higher specificity in selected lesion types suggests a practical advantage in reducing false-positive findings and potentially limiting unnecessary biopsies. In contrast, MRI maintains a critical role in complex diagnostic scenarios due to its superior sensitivity, soft tissue contrast, and lesion conspicuity, particularly for subtle, diffuse, or infiltrative disease patterns. These distinctions highlight that CEM is not merely an alternative to MRI, but a complementary modality with distinct strengths that can be strategically utilized within tailored imaging pathways.

Importantly, the review identifies three interrelated dimensions that govern modality selection: diagnostic accuracy, workflow feasibility, and economic efficiency. Evidence indicates that while both CEM and MRI improve lesion characterization and surgical planning, their implementation is shaped by practical considerations, including contrast administration protocols, examination time, accessibility, and institutional expertise. CEM offers notable advantages in terms of shorter acquisition time, greater accessibility, and easier integration into existing mammography workflows, making it particularly suitable for settings with limited MRI availability. However, its integration into screening and diagnostic pathways requires careful patient selection and standardized protocols to mitigate the risk of increased false-positive rates. From a health systems perspective, both modalities demonstrate potential cost-effectiveness by improving diagnostic precision and reducing downstream interventions. Nevertheless, cost–benefit outcomes are highly dependent on local resource availability, patient population characteristics, and the specific clinical use case (e.g., screening versus preoperative staging). This underscores the need for context-specific implementation strategies rather than universal recommendations.

A key contribution of this review lies in the identification of clinical scenarios where CEM provides the greatest added value, particularly in preoperative assessment, evaluation of multifocal disease, and cases where MRI is contraindicated or unavailable. At the same time, the review highlights persistent gaps, including limited large-scale prospective studies, heterogeneity in imaging protocols, and insufficient integration of emerging technologies such as artificial intelligence for standardized interpretation. Importantly, the decision to include only studies published in 2025 was intentional and aligned with the objective of providing a focused synthesis of the most current evidence in this rapidly evolving field. As CEM continues to advance, recent studies are more likely to reflect up-to-date technological developments, optimized imaging protocols, and contemporary comparisons with MRI. This approach is supported by recent literature emphasizing that recency-focused systematic reviews are appropriate for capturing current clinical trends and technological advancements, particularly in imaging and precision medicine domains [7,8,16]. While this approach resulted in a relatively small dataset (*n* = 20), it enhances the timeliness and clinical relevance of the findings. The PRISMA-guided selection process ensured that all included studies were of high quality and directly relevant. Nonetheless, this criterion may have excluded earlier high-quality research, and this limitation should be considered when interpreting the results. Future reviews may benefit from a broader temporal scope to provide a more comprehensive longitudinal perspective.

This review has several limitations that should be considered when interpreting the findings. The review was restricted to studies indexed in Scopus and PubMed and published in the English language. Although these restrictions may have introduced publication and language bias, the selection of these databases was intentional. Scopus was chosen for its broad multidisciplinary coverage and rigorous indexing standards, whereas PubMed was selected due to its established role as a leading source of biomedical and clinical research. Together, these databases provide extensive coverage of high-quality literature relevant to breast imaging and diagnostic medicine. Nevertheless, potentially relevant studies indexed in other databases, such as Embase, Web of Science, and the Cochrane Library, as well as studies published in languages other than English, may have been excluded. Consequently, the findings should be interpreted within the context of these search restrictions. In addition, some included studies provided limited methodological detail or incomplete reporting, which may have constrained the depth of data extraction and thematic synthesis. As a result, the findings should be regarded as indicative rather than definitive, particularly when considering their clinical applicability. Another limitation relates to the quality assessment framework employed in this review. The adopted tool was originally developed for structured review methodology rather than clinical diagnostic research. Although it enabled a consistent appraisal across heterogeneous study designs, its ability to discriminate methodological quality among diagnostic imaging studies may be limited. Future reviews focusing primarily on diagnostic accuracy evidence may benefit from the use of design-specific risk-of-bias assessment instruments, such as QUADAS-2.

Furthermore, the included studies exhibited substantial methodological heterogeneity, encompassing diagnostic accuracy studies, screening trials, implementation research, qualitative investigations, and economic evaluations. This diversity, while valuable for providing a broad overview of current evidence, precluded quantitative pooling of results and formal meta-analysis. Future research should prioritize standardized methodological reporting, comprehensive full-text accessibility, multicenter prospective investigations, and quantitative evidence synthesis to enhance the robustness, comparability, and clinical interpretability of findings in this rapidly evolving field. In conclusion, optimal breast imaging should not rely on a single modality but instead adopt a stratified, multimodal approach that aligns imaging selection with clinical objectives, lesion characteristics, and resource considerations. Future research should prioritize longitudinal outcome-based studies, multicenter validation, and the integration of advanced analytical tools to further refine diagnostic pathways. Such efforts will be essential to fully leverage the complementary strengths of CEM and MRI, ultimately enhancing diagnostic accuracy, supporting individualized clinical decision-making, and improving the overall efficiency of breast cancer care.

## Figures and Tables

**Table 2:** The selection criterion in searching.

Criterion	Inclusion	Exclusion
Language	English	Non-English
Timeline	2025	<2025
Literature type	Journal (Article)	Conference, Book, Review
Publication stage	Final	In-press
Subject	Medicine	Besides Medicine

**Table 3:** Number and details of primary studies database.

PS	Authors	Title	Year	Journal
1	Gouda et al. [30]	Contrast-enhanced mammography compared to MRI for the diagnosis of multifocal and multicentric breast cancer: preoperative evaluation	2025	Egyptian Journal of Radiology and Nuclear Medicine
2	Haggag et al. [31]	Could new mammography techniques be an alternative for dynamic contrast-enhanced breast MRI in the diagnosis and preoperative staging of breast lesions?	2025	Egyptian Journal of Radiology and Nuclear Medicine
3	ElSayad et al. [32]	Comparative analysis of digital mammography and contrast-enhanced mammography in diagnosing suspicious breast calcifications: implications for surgical decision-making	2025	Egyptian Journal of Radiology and Nuclear Medicine
4	Fueger et al. [33]	Cost-effectiveness of contrast-enhanced breast MRI in suspicious mammographic microcalcifications	2025	Insights into Imaging
5	Wang et al. [34]	Head-to-head comparison of contrast-enhanced mammography and MRI in assessing the tumor response to neoadjuvant therapy in breast cancer: a prospective, multireader study	2025	Radiologia Medica
6	Ma et al. [35]	Comparison of contrast-enhanced cone-beam breast CT, MRI, and mammography for breast cancer characterization	2025	European Radiology
7	Azcona Sáenz et al. [36]	Preoperative estimation of the pathological breast tumor size in architectural distortions: a comparison of DM, DBT, US, CEM, and MRI	2025	European Radiology
8	Bülüç et al. [37]	Quantitative analysis of breast lesions on contrast-enhanced mammography and comparison with histopathological results	2025	Acta Radiologica
9	Clauser et al. [38]	Contrast-Enhanced Digital Breast Tomosynthesis Compared With Contrast-Enhanced Mammography and Magnetic Resonance Imaging in the Assessment of Breast Lesions: A Pilot Study	2025	Investigative Radiology
10	Santonocito et al. [39]	A head-to-head comparison of breast lesion’s conspicuity at contrast-enhanced mammography and contrast-enhanced MRI	2025	European Radiology
11	Berg et al. [40]	Screening for Breast Cancer with Contrast-enhanced Mammography as an Alternative to MRI: SCEMAM Trial Results	2025	Radiology
12	Dashevsky et al. [41]	Contrast-Enhanced Mammography Implementation: Early Struggles and Successes	2025	Journal of Breast Imaging
13	Hua et al. [42]	Diagnostic performance of the Kaiser score for contrast-enhanced mammography and magnetic resonance imaging in breast masses: A Comparative Study	2025	Academic Radiology
14	Johansson Lipinski et al. [43]	Contrast-Enhanced Digital Mammography for the Diagnosis and Determination of the Extent of Disease in Invasive Lobular Carcinoma: Our Experience and Literature Review	2025	Journal of Computer Assisted Tomography
15	Horvat et al. [44]	Comparison of Contrast-enhanced Mammography and Low-Energy Imaging with or without Supplemental Whole-Breast US in Breast Cancer Detection	2025	Radiology
16	Ferrara et al. [45]	Background parenchymal enhancement in CEM and MRI: Is there always a high agreement?	2025	European Journal of Radiology
17	Singla et al. [46]	Does Contrast-Enhanced Mammography Outperform Digital Breast Tomosynthesis for Detection and Characterization of Breast Lesions or Vice Versa?	2025	Indian Journal of Surgical Oncology
18	Morris et al. [47]	Initial Attempted Contrast-Enhanced Mammography–Guided Biopsy for Suspicious Breast MRI Findings: A Single Institution’s Experience	2025	American Journal of Roentgenology
19	Aloufi et al. [48]	Accuracy of Abbreviated Breast MRI in Diagnosing Breast Cancer in Women with Dense Breasts Compared with Standard Imaging Modalities	2025	Saudi Journal of Medicine and Medical Sciences
20	Nicosia et al. [49]	Influence of Breast Density and Menopausal Status on Background Parenchymal Enhancement in Contrast-Enhanced Mammography: Insights from a Retrospective Analysis	2025	Cancers

**Table 4:** Quality assessment results of selected primary studies.

PS	QA1	QA2	QA3	QA4	QA5	QA6	Total Mark	%
PS1	Y	Y	P	Y	Y	N	4.5	75.0
PS2	Y	Y	P	Y	Y	N	4.5	75.0
PS3	Y	Y	P	Y	Y	N	4.5	75.0
PS4	Y	Y	P	Y	P	N	4.0	66.7
PS5	Y	Y	P	Y	Y	N	4.5	75.0
PS6	Y	Y	P	Y	Y	N	4.5	75.0
PS7	Y	Y	P	Y	Y	N	4.5	75.0
PS8	Y	Y	P	Y	Y	N	4.5	75.0
PS9	Y	Y	P	Y	Y	N	4.5	75.0
PS10	Y	Y	P	Y	Y	N	4.5	75.0
PS11	Y	Y	P	Y	Y	P	4.5	75.0
PS12	Y	Y	P	Y	Y	N	4.5	75.0
PS13	Y	Y	P	Y	Y	N	4.5	75.0
PS14	Y	Y	P	Y	Y	N	4.5	75.0
PS15	Y	Y	P	Y	Y	N	4.5	75.0
PS16	Y	Y	P	Y	Y	N	4.5	75.0
PS17	Y	Y	P	Y	Y	N	4.5	75.0
PS18	Y	Y	P	Y	P	N	4.0	66.7
PS19	Y	Y	P	Y	Y	N	4.5	75.0
PS20	Y	Y	P	Y	Y	N	4.5	75.0

**Table 5:** Summary of clinical applications of CEM and MRI.

Authors	Modality Studied	Clinical Application	Sample Size and Patient Cohort	Key Findings & Diagnostic Performance	Impact on Clinical Decision-Making
Gouda et al. [30]	CEM, MRI	Preoperative staging of multifocal/multicentric breast cancer (MMBC)	60 patients with suspected MMBC	CEM Sensitivity: 97% CEM Accuracy: 95% (MRI: 94%) CEM Specificity: 67% (MRI: 33%)	CEM is a promising alternative to MRI for MMBC diagnosis with significantly higher specificity.
Haggag et al. [31]	CEM, MRI, DBT	Diagnosis and preoperative staging	40 patients (53 lesions); BI-RADS 3–5	MRI showed highest sensitivity and accuracy. Combined CEM + DBT increased sensitivity to approach MRI levels.	Combined CEM and DBT can be a reasonable alternative to MRI for assessing lesion multiplicity.
ElSayad et al. [32]	CEM, DM	Evaluation of suspicious calcifications and surgical decision-making	54 lesions (35 malignant, 19 benign)	CEM Accuracy: 89% (DM: 78%) CEM Size Correlation: r = 0.86 (DM: r = 0.588).	CEM identified multicentricity missed by DM in dense breasts, enhancing surgical planning.
Fueger et al. [33]	MRI	Management of BI-RADS 4 microcalcifications	Decision analysis/Markov modeling	MRI strategy: $56,918, 2.932 QALYs Stereotactic biopsy: $56,898, 2.930 QALYs.	MRI is a cost-effective non-invasive alternative to stereotactic biopsy for risk stratification.
Wang et al. [34]	CEM, MRI	Monitoring response to neoadjuvant therapy (NAT)	57 patients before and after NAT	CEM Accuracy: 75.4% (MRI: 86.0%) CEM Sensitivity: 77.8% (MRI: 71.4%).	Both modalities slightly overestimate residual tumor; CEM is an acceptable alternative for NAT monitoring.
Ma et al. [35]	CE-CBBCT, MRI, Mammography	Breast cancer characterization	207 patients (214 malignant lesions)	Perfect agreement between CE-CBBCT and MRI for lesion type (Kappa = 0.865).	CE-CBBCT combines morphology, hemodynamics, and calcifications, facilitating BI-RADS standardization.
Azcona Sáenz et al. [36]	CEM, MRI, DM, DBT, US	Preoperative tumor size in architectural distortions (ADs)	59 patients (63 ADs)	Size concordance: CEM (75%), MRI (67.6%) DM/DBT overestimate size if thin spicules are included.	CEM, MRI, and DBT (excluding spicules) are most accurate for staging and treatment planning in ADs.
Bülüç et al. [37]	CEM	Quantitative differentiation of benign vs. malignant lesions	164 patients (170 lesions)	Infiltrating tumors showed higher relative signal density than benign lesions (*p* < 0.001).	CEM effectively distinguishes benign from malignant lesions using signal density.
Clauser et al. [38]	CE-DBTp, CEM, MRI	Assessment of suspicious findings	84 patients (91 lesions)	MRI Accuracy: 94.5% (CE-DBTp/CEM: 76.9–86.8%) CE-DBTp showed higher conspicuity than CEM.	MRI remains the most accurate; CE-DBTp improves reader confidence over standard CEM.
Santonocito et al. [39]	CEM, MRI	Comparison of lesion conspicuity	388 patients (462 lesions)	Conspicuity significantly higher for MRI (AUC 0.670–0.723) 44.7% of CEM lesions showed no enhancement.	Low conspicuity of benign lesions on CEM may reduce false-positive results in clinical practice.
Berg et al. [40]	CEM, DBT	Screening in women at elevated risk	601 women eligible for screening MRI	CEM Incremental Cancer Detection Rate: 10/1000 FPR increased by 13.4% with CEM addition.	Adding CEM to DBT significantly increases detection of early-stage, node-negative cancers.
Dashevsky et al. [41]	CEM	Clinical implementation and workflow	10 breast imaging radiologists	Not Reported (Focus group findings on workflow, contrast, and billing).	Implementation requires training for contrast reactions and addressing billing/FDA hurdles.
Hua et al. [42]	CEM, MRI	Kaiser Score (KS) for mass characterization	275 patients with enhanced masses	KS-CEM Accuracy: AUC 0.932 (MRI: AUC 0.876) Edema did not improve CEM performance.	KS for CEM provides high diagnostic accuracy comparable to MRI for distinguishing breast masses.
Johansson Lipinski et al. [43]	CEM, DBT	Diagnosis/staging of Invasive Lobular Carcinoma (ILC)	24 examinations in elderly patients	CEM size strongly correlated to pathology (r = 0.94–0.99).	CEM is highly accurate for assessing ILC extent, especially when MRI is contraindicated.
Horvat et al. [44]	CEM, LE, US	Screening performance	468 participants	Cancer Detection Rate: CEM (17.1/1000), LE+US (8.5/1000), LE (2.1/1000).	CEM significantly improves cancer detection compared to LE images alone or LE supplemented with US.
Ferrara et al. [45]	CEM, MRI	Background Parenchymal Enhancement (BPE) agreement	343 patients	Fair to moderate BPE agreement between modalities (Kappa 0.342–0.432).	CEM BPE is influenced by compression force and breast density, potentially impacting interpretation.
Singla et al. [46]	CEM, DBT	Detection and characterization of lesions	58 women (62 lesions)	CEM Sensitivity: 100% (DBT: 95%) CEM Specificity: 88% (DBT: 77.8%).	CEM provided superior delineation of disease extent and identified contralateral lesions, altering management.
Morris et al. [47]	CEM, MRI	Biopsy guidance for suspicious findings	Not Reported	Not Reported	CEM was utilized for guided biopsy of suspicious findings originally identified on MRI.
Aloufi et al. [48]	ABMRI, Mammography, DBT, US	Dense breast screening/diagnosis	55 women with dense breasts	ABMRI Sensitivity: 94.7% (Mammography: 84.2%) ABMRI AUC: 0.751 (Mammography: 0.643).	Abbreviated MRI (ABMRI) is more accurate than mammography/DBT for women with dense breast tissue.
Nicosia et al. [49]	CEM	Impact of density and menopause on BPE	116 patients with confirmed BC	Higher BPE linked to high density (*p* < 0.001) and pre-menopausal status (*p* = 0.029).	BPE intensity was not associated with tumor grade, suggesting BPE as a standalone risk biomarker.

## Data Availability

No new data were created or analyzed in this study. Data sharing is not applicable to this article.
